# Supplementary material for: Sae2/CtIP prevents R-loop accumulation in eukaryotic cells
Source: eLife. 2018 Dec 7;7:e42733. doi: 10.7554/eLife.42733 (PMC6296784; doi:10.7554/eLife.42733)
Supplement: Supplementary file 1. [file elife-42733-supp1.docx]

| Strain List | |  | |  | |  | |  | |
| --- | --- | --- | --- | --- | --- | --- | --- | --- | --- |
|  | |  | |  | |  | |  |  |
| name | | genotype | | plasmid | | figures | | ref |  |
| BY4741 (TP1158) | | *MATa his3Δ1 leu2Δ0 met15Δ0 ura3Δ0* | |  | | 1D, 2A, 2B | | (*1*) |  |
| TP6986 | | *MATa his3Δ1 leu2Δ0 met15Δ0 ura3Δ0* | | pRS425 (*2*) | | 1A, 2E | | this study |  |
| TP2012 | | *MATa his3Δ1 leu2Δ0 met15Δ0 ura3Δ0 sae2::KMX* | |  | | 1D, 2A, 2B | | (*3*) |  |
| TP3540 | | *MATa his3Δ1 leu2Δ0 met15Δ0 ura3Δ0 sae2::KMX* | | pRS425 (*2*) | | 1A, 2E | | this study |  |
| TP7053 | | *MATa his3Δ1 leu2Δ0 met15Δ0 ura3Δ0 sae2::KMX* | | *PCF11* in pRS425 (Steve Hanes) | | 1A | | this study |  |
| TP7190 | | *MATa his3Δ1 leu2Δ0 met15Δ0 ura3Δ0 sae2::KMX* | | pTP3249 (*SSU72* in pRS425) | | 1A | | this study |  |
| TP7191 | | *MATa his3Δ1 leu2Δ0 met15Δ0 ura3Δ0 sae2::KMX* | | pTP3498 (*RTT103* in pRS425) | | 1A | | this study |  |
| TP7201 | | *MATa his3Δ1 leu2Δ0 met15Δ0 ura3Δ0 sae2::KMX* | | pTP3500 (*SEN1* in pRS425) | | 1A, 2E | | this study |  |
| TP7204 | | *MATa his3Δ1 leu2Δ0 met15Δ0 ura3Δ0 sae2::KMX* | | pTP3564 (*sen1-G1747D* in pRS425) | | 1A | | this study |  |
| TP7203 | | *MATa his3Δ1 leu2Δ0 met15Δ0 ura3Δ0 sae2::KMX* | | pTP3563 (*sen1-R302W* in pRS425) | | 1A | | this study |  |
| W1588-4C (TP1733) | | *MATa RAD5 leu2-3,112 trp1-1 ura3-1 can1-100 his3-11,15 ade2-1* | |  | |  | | Hannah Klein |  |
| TP7851 | | *MATa RAD5 leu2-3,112 trp1-1 ura3-1 can1-100 his3-11,15 ade2-1* | | pRS425 (*2*) | | 1B | | this study |  |
| TP7692 | | *MATa RAD5 leu2-3,112 trp1-1 ura3-1 can1-100 his3-11,15 ade2-1 sae2::URA3* | |  | |  | | this study |  |
| TP7852 | | *MATa RAD5 leu2-3,112 trp1-1 ura3-1 can1-100 his3-11,15 ade2-1 sae2::URA3* | | pRS425 (*2*) | | 1B | | this study |  |
| TP7819 | | *MATa RAD5 leu2-3,112 trp1-1 ura3-1 can1-100 his3-11,15 ade2-1 sae2::URA3* | | pTP3500 (*SEN1* in pRS425) | | 1B | | this study |  |
| LSY1397 (TP1754) | | *MATa RAD5 leu2-3,112 trp1-1 ura3-1 can1-100 his3-11,15 ade2-1 mre11-H125N* | |  | |  | | Lorraine Symington |  |
| TP7757 | | *MATa RAD5 leu2-3,112 trp1-1 ura3-1 can1-100 his3-11,15 ade2-1 mre11-H125N* | | pRS425 (*2*) | | 1B | | this study |  |
| TP7758 | | *MATa RAD5 leu2-3,112 trp1-1 ura3-1 can1-100 his3-11,15 ade2-1 mre11-H125N* | | pTP3500 (*SEN1* in pRS425) | | 1B | | this study |  |
| TP7751 | | *MATa RAD5 leu2-3,112 trp1-1 ura3-1 can1-100 his3-11,15 ade2-1 mre11-H125N sae2::URA3* | |  | |  | |  |  |
| TP7820 | | *MATa RAD5 leu2-3,112 trp1-1 ura3-1 can1-100 his3-11,15 ade2-1 mre11-H125N sae2::URA3* | | pRS425 (*2*) | | 1B | | this study |  |
| TP7794 | | *MATa RAD5 leu2-3,112 trp1-1 ura3-1 can1-100 his3-11,15 ade2-1 mre11-H125N sae2::URA3* | | pTP3500 (*SEN1* in pRS425) | | 1B | | this study |  |
| 1971 (TP7480) | | *MATalpha leu2-3,112 ura3-52 pep4-3* | |  | | 1C | | (*4*) |  |
| TP8347 | | *MATalpha leu2-3,112 ura3-52 pep4-3 sae2::URA3* | |  | | 1C | | this study |  |
| FWY1 (TP7481) | | *MATalpha leu2-3,112 ura3-52 pep4-3 sen1-1* | |  | | 1C | | (*4*) |  |
| TP8348 | | *MATalpha leu2-3,112 ura3-52 pep4-3 sen1-1 sae2::URA3* | |  | | 1C | | this study |  |
| YAEH255 (TP7483) | | BY4741 *rnh1::KMX rnh201::NatMx6* | |  | | 1D | | *6*)(*5*) |  |
| TP7524 | | BY4741 *rnh1::KMX rnh201::NatMx6 sae2::URA3* | |  | | 1D | | this study |  |
| TP2536 | | *MATa RAD5 leu2-3,112 trp1-1 ura3-1 can1-100 his3-11,15 ade2-1 sae2::KMX* | |  | |  | | this study |  |
| TP2552 | | *MATa RAD5 leu2-3,112 trp1-1 ura3-1 can1-100 his3-11,15 ade2-1 sae2::KMX* | | pRS316 | | 1E | | this study |  |
| TP7221 | | *MATa RAD5 leu2-3,112 trp1-1 ura3-1 can1-100 his3-11,15 ade2-1 sae2::KMX* | | wild-type RNH1 in pBY011 (PlasmID ID DNA Resource Core) | | 1E | | this study |  |
| TP3352 | | *MATa his3Δ1 leu2Δ0 met15Δ0 ura3Δ0 sae2::KMX* | | wild-type Flag-*SAE2* in pRS425 (*6*) | | 2C, 2D | | this study |  |
| yJC 2695  (TP7118) | | *BY4733 Rpb2-HTB* | |  | |  | | Jeff Corden (*7*) |  |
| TP7130 | | *BY4733 Rpb2-HTB* | | pRS425 (*2*) | | 2E,3A,B | | this study |  |
| TP7297 | | *BY4733 Rpb2-HTB sae2::URA3* | |  | |  | | this study |  |
| TP7327 | | *BY4733 Rpb2-HTB sae2::URA3* | | pRS425 (*2*) | | 2E,3A,B | | this study |  |
| TP7330 | | *BY4733 Rpb2-HTB sae2::URA3* | | pTP3500 (*SEN1* in pRS425) | | 2E,3A,B | | this study |  |
| YJC 2671 | | BY4733 *Sen1-HTB* | |  | | 3C | | Jeff Corden (8) |  |
| TP7296 | | BY4733 *Sen1-HTB sae2::URA3* | |  | | 3C | | this study |  |
| TP7190 | | *MATa his3Δ1 leu2Δ0 met15Δ0 ura3Δ0 sae2::KMX* | | pTP3249 (*SSU72* in pRS425) | | 1 - S1 | | this study |  |
| TP7191 | | *MATa his3Δ1 leu2Δ0 met15Δ0 ura3Δ0 sae2::KMX* | | pTP3498 (*RTT103* in pRS425) | | 1 - S1 | | this study |  |
| TP7200 | | *MATa his3Δ1 leu2Δ0 met15Δ0 ura3Δ0 sae2::KMX* | | pTP3509 (*YSH1* in pRS425) | | 1 - S1 | | this study |  |
| TP7205 | | *MATa his3Δ1 leu2Δ0 met15Δ0 ura3Δ0 sae2::KMX* | | pRS425-NRD1(Steve Hanes) | | 1 - S1 | | this study |  |
| *YMV80 (*TP6028) | | *hml*Δ::*ADE1 mat*aΔ::hisG *hmr*Δ::*ADE1 leu2-cs ade3*::*GAL*::*HO ade1 lys5 ura3-52* | | pRS425 (*2*) | | 1-S2 | | S.E. Lee, J. Haber (9) |  |
| TP7703 | | *YMV80 sae2::URA3* | | pRS425 (*2*) | | 1-S2 | | this study |  |
| TP6030 | | *YMV80 sae2::URA3* | | wild-type Flag-*SAE2* in pRS425 (*6*) | | 1-S2 | | this study |  |
| TP7841 | | *YMV80 sae2::URA3* | | pTP3500 (*SEN1* in pRS425) | | 1-S2 | | this study |  |

**References**

1. Brachmann, C. B., A. Davies, G. J. Cost, E. Caputo, J. Li, P. Hieter, and J. D. Boeke. “Designer Deletion Strains Derived from Saccharomyces Cerevisiae S288C: A Useful Set of Strains and Plasmids for PCR-Mediated Gene Disruption and Other Applications.” *Yeast* 14 (1998): 115–32.

2. Christianson, T. W., R. S. Sikorski, M. Dante, J. H. Shero, and P. Hieter. “Multifunctional Yeast High-Copy-Number Shuttle Vectors.” *Gene* 110 (1992): 119–22.

3. Shoemaker, Daniel D., Deval A. Lashkari, Don Morris, Mike Mittmann, and Ronald W. Davis. “Quantitative Phenotypic Analysis of Yeast Deletion Mutants Using a Highly Parallel Molecular Bar–coding Strategy.” *Nature Genetics* 14, no. 4 (1996): 450–56.

4. Ursic, D., K. L. Himmel, K. A. Gurley, F. Webb, and M. R. Culbertson. “The Yeast SEN1 Gene Is Required for the Processing of Diverse RNA Classes.” *Nucleic Acids Research* 25, no. 23 (1997): 4778–85.

5. El Hage, Aziz, Shaun Webb, Alastair Kerr, and David Tollervey. “Genome-Wide Distribution of RNA-DNA Hybrids Identifies RNase H Targets in TRNA Genes, Retrotransposons and Mitochondria.” *PLoS Genetics* 10, no. 10 (2014): e1004716.

6. Kim, H. S., S. Vijayakumar, M. Reger, J. C. Harrison, J. E. Haber, C. Weil, and J. H. Petrini. “Functional Interactions between Sae2 and the Mre11 Complex.” *Genetics* 178 (2008): 711–23.

7. Schaughency, Paul, Jonathan Merran, and Jeffry L. Corden. “Genome-Wide Mapping of Yeast RNA Polymerase II Termination.” Edited by Nick J. Proudfoot. *PLoS Genetics* 10, no. 10 (2014): e1004632.

8. Creamer, Tyler J., Miranda M. Darby, Nuttara Jamonnak, Paul Schaughency, Haiping Hao, Sarah J. Wheelan, and Jeffry L. Corden. “Transcriptome-Wide Binding Sites for Components of the Saccharomyces Cerevisiae Non-Poly(A) Termination Pathway: Nrd1, Nab3, and Sen1.” *PLoS Genetics* 7, no. 10 (2011): e1002329.

9. Vaze, M. B. *et al.* Recovery from checkpoint-mediated arrest after repair of a double-strand break requires Srs2 helicase. *Mol Cell* **10,** 373–85 (2002).
